# Non-Alcoholic Steatohepatitis: A Review of Its Mechanism, Models and Medical Treatments

**DOI:** 10.3389/fphar.2020.603926

**Published:** 2020-12-03

**Authors:** Cheng Peng, Alastair G. Stewart, Owen L. Woodman, Rebecca H. Ritchie, Cheng Xue Qin

**Affiliations:** ^1^ Drug Discovery Biology, Monash Institute of Pharmaceutical Sciences, Melbourne, VIC, Australia; ^2^ Baker Heart & Diabetes Institute, Melbourne, VIC, Australia; ^3^ Department of Pharmacology and Therapeutics, University of Melbourne, Melbourne, VIC, Australia; ^4^ Australian Research Council, Centre for Personalised Therapeutics Technologies, Lancaster, CBR, Australia

**Keywords:** animal models, pharmacological treatments, metabolic syndrome, obesity, non-alcoholic fatty liver disease, non-alcoholic steatohepatitis, steatosis

## Abstract

Non-alcoholic steatohepatitis (NASH) develops from non-alcoholic fatty liver disease (NAFLD). Currently, around 25% of the population is estimated to have NAFLD, and 25% of NAFLD patients are estimated to have NASH. NASH is typically characterized by liver steatosis inflammation, and fibrosis driven by metabolic disruptions such as obesity, diabetes, and dyslipidemia. NASH patients with significant fibrosis have increased risk of developing cirrhosis and liver failure. Currently, NASH is the second leading cause for liver transplant in the United States. More importantly, the risk of developing hepatocellular carcinoma from NASH has also been highlighted in recent studies. Patients may have NAFLD for years before progressing into NASH. Although the pathogenesis of NASH is not completely understood, the current “multiple-hits” hypothesis suggests that in addition to fat accumulation, elevated oxidative and ER stress may also drive liver inflammation and fibrosis. The development of clinically relevant animal models and pharmacological treatments for NASH have been hampered by the limited understanding of the disease mechanism and a lack of sensitive, non-invasive diagnostic tools. Currently, most pre-clinical animal models are divided into three main groups which includes: genetic models, diet-induced, and toxin + diet-induced animal models. Although dietary models mimic the natural course of NASH in humans, the models often only induce mild liver injury. Many genetic and toxin + diet-induced models rapidly induce the development of metabolic disruption and serious liver injury, but not without their own shortcomings. This review provides an overview of the “multiple-hits” hypothesis and an evaluation of the currently existing animal models of NASH. This review also provides an update on the available interventions for managing NASH as well as pharmacological agents that are currently undergoing clinical trials for the treatment of NASH.

## Introduction

First discovered in 1980, non-alcoholic steatohepatitis (NASH) is a type of fatty liver disease characterized by excessive liver fat accumulation, hepatic inflammation and fibrosis (Ludwig et al., 1980; Kleiner et al., 2005; Diehl and Day, 2017). NASH is falls within the large, overarching theme of non-alcoholic fatty liver disease (NAFLD) which encompasses varying degrees of liver injury (Friedman et al., 2018a). NASH is histologically distinct from a simple fatty liver, where there is only an accumulation of fat without the presence of inflammation and fibrosis (Brunt et al., 2011).

## Epidemiology of Non-Alcoholic Steatohepatitis

**FIGURE 1:**
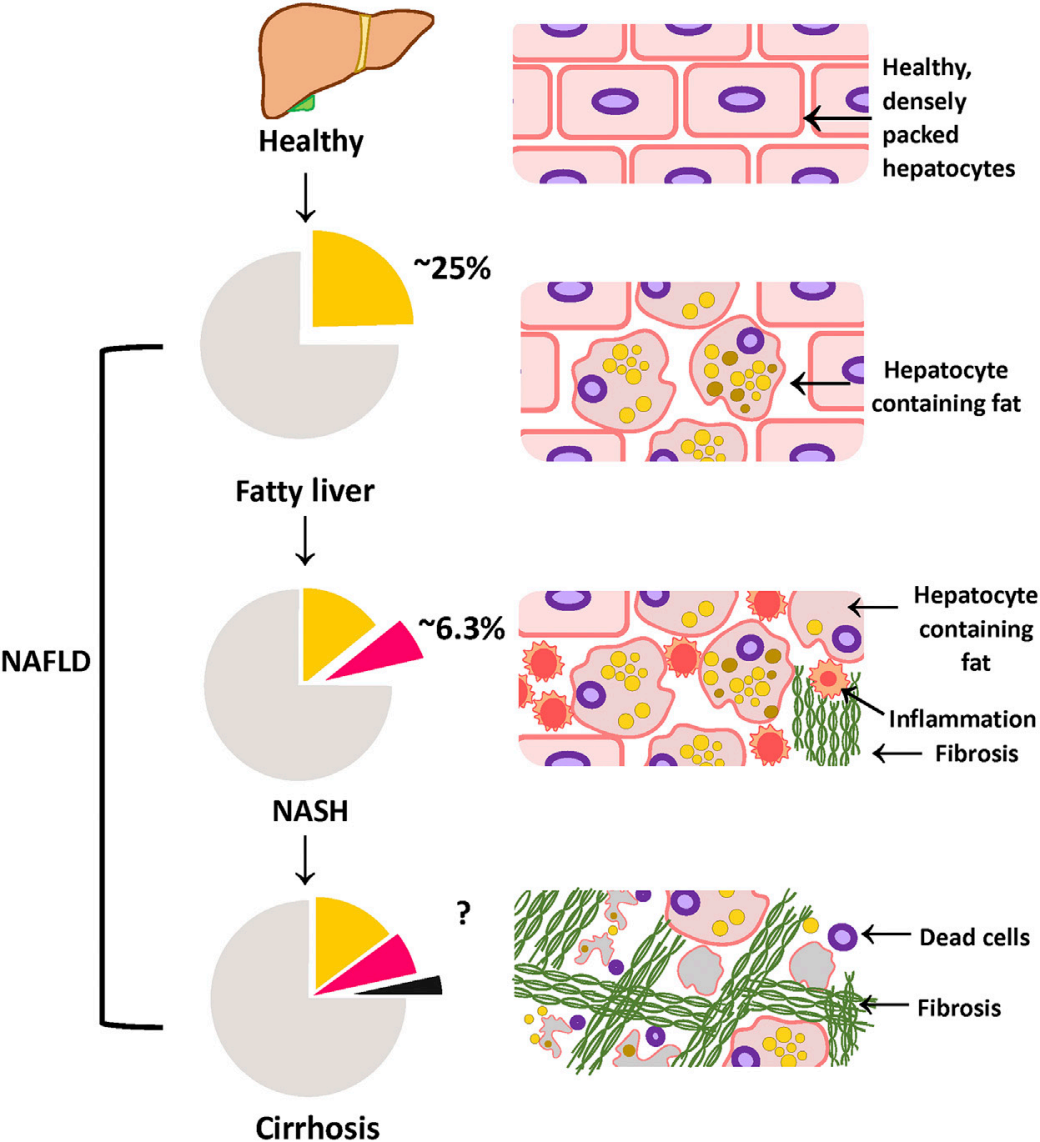
Different phases of NAFLD: progressing from healthy to cirrhosis NAFLD represents a spectrum of fatty liver diseases ranging from fatty liver to cirrhosis. Approximately 25% of the population worldwide is estimated to have fatty liver, characterized by more than 5% fat accumulating in the liver. If left untreated, fatty liver can progress onto the more severe form; NASH, defined by severe liver injury and inflammation in addition to fat. Currently, a further 25% of the NAFLD population is estimated to have NASH (which is roughly calculated to be 6.3% of the population). NASH patients are estimated to have a higher risk of developing cirrhosis, which is the extensive liver tissue scarring. Figure is designed and drawn using Inkscape (http://www.inkscape.org/.).

The growing epidemics of obesity, dyslipidemia and insulin resistance serve as major risk factors for the development of NASH (Saklayen, 2018). Epidemiological studies show that roughly 82% of NASH patients are obese, 83% exhibit hyperlipidemia and 48% are diagnosed with type 2 diabetes (Younossi et al., 2016b). NAFLD tends to be more prevalent in middle-aged to elderly patients as older patients exhibit more characteristics of metabolic syndrome (Frith et al., 2009; Williams et al., 2011). Nevertheless, NAFLD can also be diagnosed in children/adolescences who are as young as 13 years old (Goyal and Schwimmer, 2016). According to the survey conducted by the National Health and Nutrition Examination in the United States, the incidence of NAFLD in adolescent and young adults (aged 19–35) have risen by approximately 2.5 times in the last 20 years (Welsh et al., 2013). More importantly, longitudinal follow-up studies suggest that adolescent diagnosed with NAFLD/NASH have increased risk of cirrhosis and mortality compared to age-matched average population (Feldstein et al., 2009; Goyal and Schwimmer, 2016; Doycheva et al., 2017). Currently, it is estimated that approximately 25% of the world population has NAFLD, and further, 20–25% of this NAFLD patient population will go on to develop NASH (Younossi et al., 2016a; Estes et al., 2018) (Figure 1). If left untreated, the risk of developing cirrhosis, and subsequently liver failure and hepatocellular carcinoma will increase and eventually causing death (Alexander et al., 2019). NASH-induced cirrhosis has been recognized as one of the fastest-growing liver diseases, and is the second greatest contributor to an indication for liver transplantation in the United States (Wong et al., 2015). Based on current trends, there will be an estimated global NAFLD incidence of 101 million by 2030, and the number of NASH cases is projected to increase to 27 million by 2030 (Estes et al., 2018). In parallel with the development and progression of obesity and type 2 diabetes a recent study highlighted that the annual health care cost associated with NAFLD in the United States was approximately US$103 billion, and €35 billion in four European countries combined (Germany, Italy, United Kingdom., and France) (Younossi et al., 2016a). These costs are estimated to rise to US$908 and €302 billion in the United States and in these European countries, respectively within 10 years (Younossi et al., 2016a). Thus, early detection, diagnosis and treatment of fatty liver disease are of paramount importance in controlling the impact of this disease.

## Diagnosis and Detection Methods

NASH itself can often be asymptomatic, although patients with a high body mass index (>25 kg/m^2^) and T2DM features such as hyperglycemia and insulin resistance are encouraged to be screened for the presence of fatty liver disease (Chalasani et al., 2012; Friedman et al., 2018a). Nevertheless, a recent population study has highlighted that NASH patients have a higher incidence of fatigue and abdominal discomfort which are shown to be correlated with hepatic lobular inflammation (Huber et al., 2019). This may be because that hepatic inflammation is associated with elevated plasma inflammatory cytokines (Ajmera et al., 2017) which creates a metabolically inflamed milieu that can negatively affect the mood (Rethorst et al., 2014; Huber et al., 2019).

Patients consuming less than the excessive alcohol intake threshold of >20–30 g/day are classified as having NAFLD, and patients who consumed above that threshold would be diagnosed as having alcoholic fatty liver disease (AFLD), typically treated by alcohol abstinence (Scaglioni et al., 2011). Although NAFLD/NASH is not a result of excessive alcohol intake, it shares many histological similarities with AFLD, such as liver steatosis and inflammation (Williams et al., 2011). Nevertheless, it might not be possible to determine whether low alcohol use contributes to the development of NAFLD/NASH (Scaglioni et al., 2011).

Elevation in the plasma of the liver enzymes alanine transaminase (ALT) and aspartate aminotransferase (AST) in a routine blood test is generally the first line of diagnosis (Kim et al., 2008; Siddiqui et al., 2018). ALT and AST are highly expressed in hepatocytes. In the event of hepatocyte necrosis, ALT and AST leak into the circulation, and are thus biomarkers for liver injury (Kew, 2000). Nevertheless, NASH patients may have normal plasma ALT/AST levels and the presence of other diseases, such as viral hepatitis, may also induce ALT and AST elevation (Dyson et al., 2014). ALT and AST are thus insufficiently specific and sensitive enough to determine the presence or severity of NASH (Friedman et al., 2018a). To confirm the presence of fatty liver, computed tomography (CT) scan or magnetic resonance imaging (MRI) can potentially be used as a non-invasive diagnostic tool to assess the percentage of fat in the liver (Friedman et al., 2018a). However, using MRI as a diagnostic tool in the clinic may not be practical due to the high cost and limited availability. Patients in rural/regional areas and/or from low socioeconomic areas would be unlikely to be able to access it (Friedman et al., 2018a). More importantly, the percentage of hepatic fat alone does not indicate the level of liver inflammation, hepatocyte damage and tissue fibrosis. Thus pathologist scoring of liver biopsy histological features remains the gold standard for determining the presence and severity of NASH (Siddiqui et al., 2018). Liver hematoxylin and eosin staining are assigned an ordinal score on a scale of 0–3 for steatosis, 0–3 for inflammation and 0–2 for ballooning hepatocytes by a panel of pathologists (Table 1). Ordinal scores of these 3 parameters are combined to give a total NAFLD activity score (NAS). In both clinical and preclinical studies, there is a general consensus that a total NAS ≥5 is classified as definitive NASH rather than a simple fatty liver disease (Kleiner et al., 2005). Nevertheless, the invasive nature of liver biopsy has made the histological diagnosis method less favored. Further studies in elucidating the molecular mechanisms of NASH to discover sensitive and highly NASH-specific biomarkers are warranted.

**TABLE 1:** NAFLD Activity Score (NAS) system with representative H&E images. The NAS system is an internationally recognized method of determining the severity of fatty liver disease (see text for references). Steatosis score represents the percentage of lipid droplets present in each field of view, inflammation score represents the number of inflammatory cell clusters (1 cluster = 1 foci) and the ballooning score is indicative of the number of hepatocytes that have altered cell structure due to excess lipid accumulation. The pathologist can give a score between 0 and 3 for each of steatosis and inflammation, and 0–2 for ballooning, based on the characteristics of the samples. Combining the scores from each of the parameters give rise to the total NAS. H&E-stained representative images are provided by our laboratory. All images were taken under ×200 magnification. Black arrows are marking the specific location of the histological features.

Features	Score	Description
Healthy 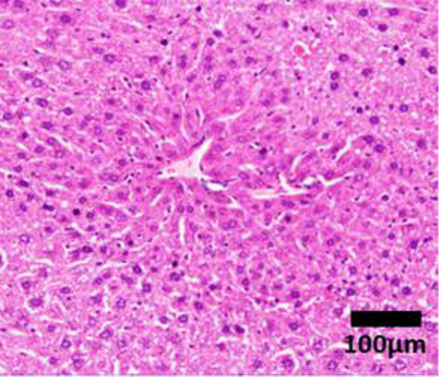	None	Healthy liver
		Hepatocytes are nicely arranged and densely packed
Steatosis 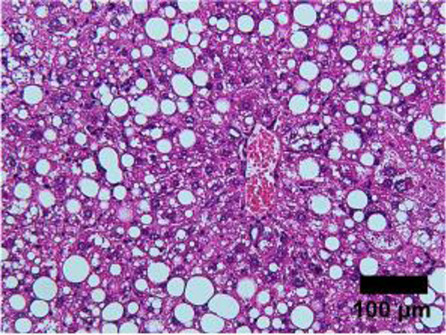	0	<5% of liver tissue (per field of view)
	1	<33% of liver tissue (per field of view)
	2	34–66% of liver tissue (per field of view)
	3	>66% of liver tissue (per field of view)
Inflammation 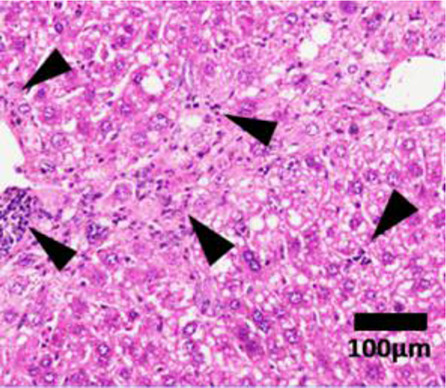	0	None
	1	1–2 foci
	2	3–4 foci
	3	>4 foci
Ballooning 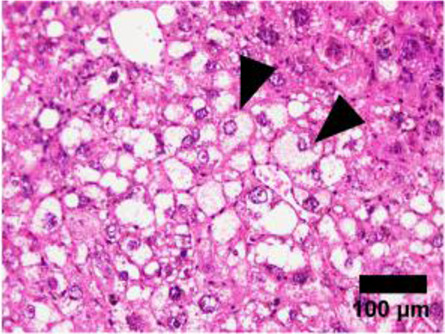	0	None
	1	Few
	2	Many

## Pathogenesis of Non-Alcoholic Steatohepatitis

**FIGURE 2:**
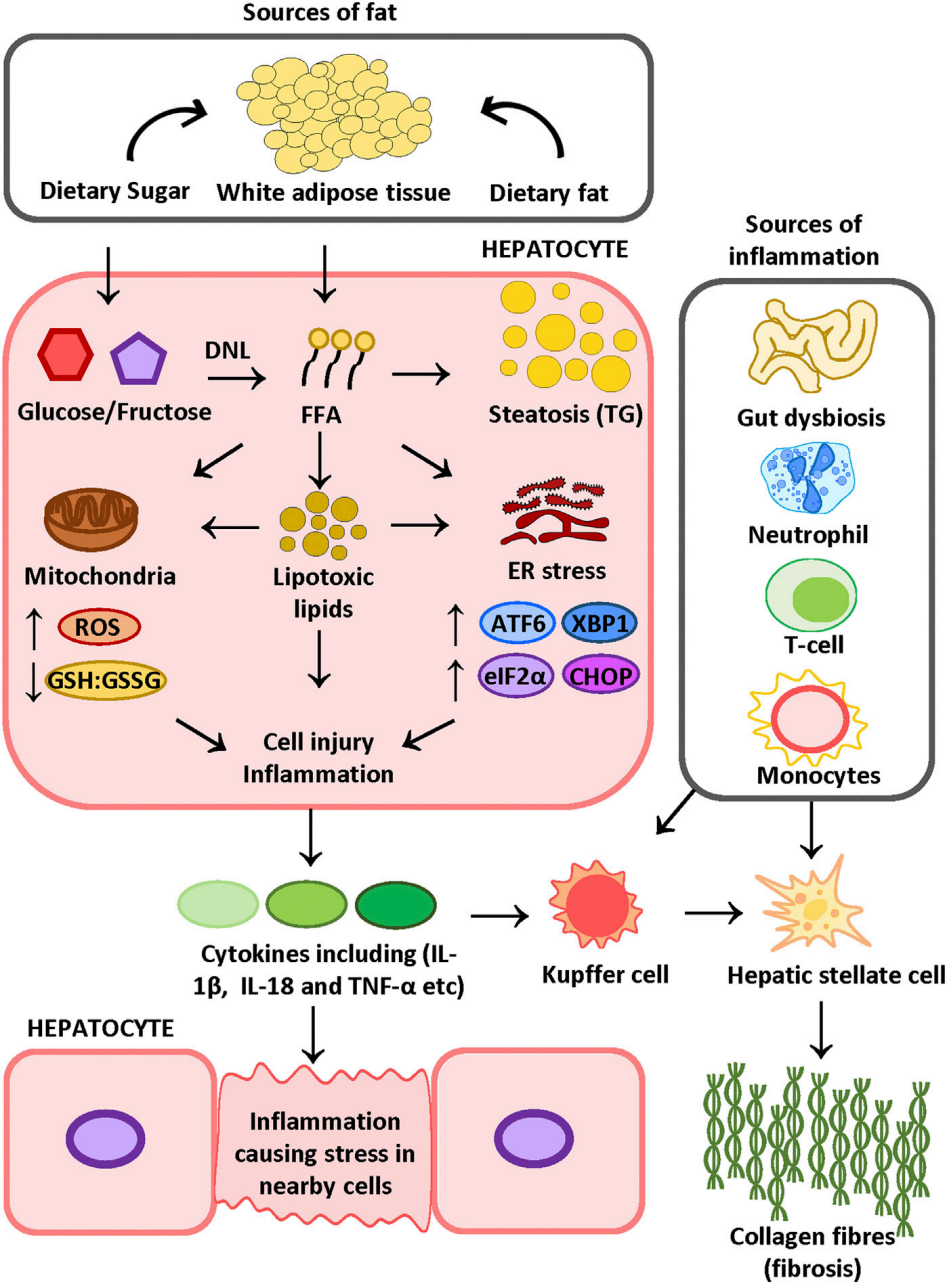
Proposed mechanism of NASH FFAs released from adipose tissue due to insulin resistance and dietary-sugar-induced DNL increase the FFA pool in the liver. FFAs can be stored as TG in the hepatocyte or be metabolized into lipotoxic lipids. Lipid mediators may induce oxidative stress and ER stress, which ultimately results in cell injury and inflammation. Cell injury induces inflammatory cell recruitment and activation. A leaky gut due to gut dysbiosis can further contribute to liver inflammation. The combination of inflammation and tissue damage triggers HSC activation and collagen deposition. FFA: free fatty acid, KC: Kupffer cell, HSC: hepatic stellate cell, TG: triglyceride, DNL: *de novo* lipogenesis, IL-18: interleukin 18, IL-1β: interleukin one beta, TNF-α: tumor necrosis factor α, ATF6: activating transcription factor 6; TXBP-1: total X-box protein-1; CHOP: C/EBP Homologous Protein, eIF2α: eukaryotic translation initiation factor 2α. Figure is designed and drawn using Inkscape (http://www.inkscape.org/.).

Day and James (1998) hypothesized the “two-hit” NASH model, i.e., that obesity, as an external stressor, can increase the accumulation of fat in the liver, but is normally not sufficient to cause inflammation and fibrosis. Hence a “second-hit” is required to further exaggerate liver injury. Recent findings on NASH shed a new light on the disease pathogenesis, shifting from the traditional “two-hit” model to a model where multiple parallel pathogenic influences are present that may act synergistically to drive the development of NASH, as indicated in Figure 2 (Buzzetti et al., 2016). Although the exact mechanism of disease pathogenesis remains to be elucidated, epidemiological studies have highlighted common metabolic comorbidities of NAFLD/NASH patients including obesity, insulin resistance and hyperlipidemia (Younossi et al., 2016a). In support of this concept, several clinical studies have suggested that NAFLD/NASH may have a role in the development of other metabolic diseases including cardiovascular diseases (Targher et al., 2010; Labenz et al., 2020) and chronic kidney disease (Kaps et al., 2020).

Recently, there has been increasing interest in the role of metabolic inflammation and the crosstalk network between liver and other organs in driving metabolic diseases (Gehrke and Schattenberg, 2020; Wang et al., 2020). The elevated pro-inflammatory gut microbes influencing the liver during gut dysbiosis may initiate and/or exacerbate hepatic inflammation (Hildebrandt et al., 2009; Ogawa et al., 2018). Moreover, the presence of disrupted metabolism such as insulin resistance in the adipose tissue is known to be associated with increased hepatic steatosis as well as hepatic macrophage activation (Rosso et al., 2019; Gehrke and Schattenberg, 2020). In addition to extrahepatic stressors, intrahepatic cellular stressors such as liver oxidative stress and endoplasmic reticulum (ER) stress are also known to be part of the multiple parallel influences mechanism which may lead to the development of NAFLD/NASH (Sanyal et al., 2001; Friedman et al., 2018a; Lebeaupin et al., 2018a). Adding to the complexity of the disease, the likelihood of NASH development also appears closely associated with genetic factors (Eslam et al., 2018).

### The Role of Genetics and Ethnicity

Familial studies indicate that children from parents with higher hepatic fat contents are more likely to develop NAFLD and cirrhosis (Schwimmer et al., 2009). Twin studies demonstrated a significantly higher intra-pair correlation between the level of liver fat and plasma ALT in monozygotic twin than dizygotic twins (Makkonen et al., 2009). In addition, genome-wide association studies have recently identified numerous genetic factors that associate strongly with the development of NAFLD. Genetic variants in genes such as transmembrane six superfamily member 2 (TM6SF2) (Mahdessian et al., 2014), glucokinase regulatory protein (GCKR) (Petta et al., 2014) and patatin-like phospholipase domain-containing-3 (PNPLA3) are found to associate with NAFLD and NASH, with PNPLA3 classified as one of the most common genetic variations (Eslam et al., 2018). Patients who have the PNPLA3 genetic polymorphism produce a truncated lipase enzyme which impedes triglyceride breakdown and subsequently reduces liver triglyceride (TG) secretion in the form of very-low-density lipoproteins (VLDL) (Dongiovanni, 2013). Interestingly, a population study conducted in the United States revealed differences in susceptibility to triglyceride accumulation, and the development of NAFLD, in different ethnic groups. The PNPLA3 I148M polymorphism is more frequently present in the Hispanic population, less in those of European descent, and lowest in African-American populations (Romeo et al., 2008). Investigators suggested that the lower frequency of the PNPLA3 I148M polymorphism offers a potential explanation for the lower prevalence of NAFLD observed in the African-American population, despite a higher rate of obesity and diabetes (Romeo et al., 2008; Chinchilla-lópez et al., 2019). Notably, gene polymorphisms alone do not completely confer full NAFLD/NASH development (Diehl and Day, 2017). Rather, genetic predisposition combined with environmental factors such as obesity, increased abdominal fat mass and excessive carbohydrate and sugar consumption may confer a higher risk of developing NASH (Davis et al., 2010).

### The Role of Obesity and Systemic Insulin Resistance 

Epidemiological studies have revealed a strong link between obesity and type 2 diabetes with the development of NASH, suggesting that an insulin-resistant *milieu* may be an important initial driving force for the development of NASH (Lomonaco et al., 2012; Younossi et al., 2016b). Nevertheless, accumulating evidence suggests that lean subjects, particularly from the Asian ethnic group, may also develop NAFLD, commonly referred to as having “lean NAFLD” (Das and Chowdhury, 2013; Feng et al., 2014; Chen et al., 2020). Lean NAFLD subjects often exhibit excess visceral white adipose tissue despite a normal BMI (25 kg/m^2^ in Caucasian and 23 kg/m^2^ in Asian populations) (Ruderman et al., 1998; Feng et al., 2014; Chen et al., 2020). Excess white adipose tissue associated with increased plasma and adipose tissue pro-inflammatory cytokines such as TNF-α and IL-6 has been reported in both patients with NAFLD and in animal models of NAFLD (Hotamisligil et al., 1993; Hotamisligil et al., 1995; Weisberg et al., 2003). In addition to contributing to chronic low-grade systemic inflammation, adipose-tissue-derived cytokines are also reported to induce systemic insulin resistance, by impeding downstream insulin signaling (Peraldi et al., 1996; Xu et al., 2003). Upon insulin binding, insulin receptor activation initiates tyrosine phosphorylation of the downstream cytosolic insulin receptor substrate (IRS) (White, 1997). This signaling cascade is transduced by IRS as it phosphorylates phosphoinositide 3-kinase (PI3K) and Akt to further elicit insulin-mediated effects (White, 1997). TNF-α inhibits insulin downstream signaling by activating c-Jun NH_2_-terminal kinase (JNK) which phosphorylates IRS-1 at Ser^307^ (Aguirre et al., 2000). As a result of insulin resistance, adipose-tissue-released free fatty acids (FFA) accumulate in the plasma (Morigny et al., 2016). Clinical studies of NAFLD patients have revealed a positive correlation between insulin resistance and elevated hepatic TGs, suggesting that the adipose-tissue-released FFAs may ultimately be taken up by the liver and metabolized into TG (Lomonaco et al., 2012). In addition to adipose-tissue-derived FFAs, increased dietary fat and carbohydrate uptake (especially fructose) can also contribute to steatosis in the liver (Faeh et al., 2005; Lomonaco et al., 2012; Lambert et al., 2014).

### The Role of Dietary Fat Intake and *de novo* Lipogenesis 

Steatosis is defined as excess TG deposition in the liver, which gives rise to lipid droplets scattered through the liver tissue (Figure 2). Notably, of all TGs found in the liver of NAFLD patients, 59% are derived from plasma FFAs, whereas 15% and 26% are derived from dietary fat and *de novo* lipogenesis, respectively (Donnelly et al., 2005). This is consistent with the role of dietary fat intake and *de novo* lipogenesis in triggering liver steatosis, in addition to adipose-tissue-derived FFAs (Lomonaco et al., 2012; Lambert et al., 2014; Luukkonen et al., 2018). In support of this, a dietary study using a stabilized isotope tracer demonstrated that human subjects on a diet rich in saturated-fat exhibited increased adipose tissue triglyceride storage and increased intrahepatic TG levels (Luukkonen et al., 2018). Moreover, long-term consumption of diets with 45–68% energy derived from fats has been reported to elevate intrahepatic TG in rodents (Wang et al., 2006; Koppe et al., 2009). Apart from direct fat uptake, TGs derived from *de novo* lipogenesis are reportedly elevated in subjects who were on a high carbohydrate diet (Faeh et al., 2005; Luukkonen et al., 2018). Dietary studies in rodents fed with a high fructose diet showed activation of the lipogenic transcription factor, sterol regulatory element-binding protein 1c (SCREBP1c), which is responsible for inducing the transcription of lipogenic enzymes for catalyzing TG synthesis (Aragno et al., 2009; Softic et al., 2016). Overall, hepatic FFA accumulation contributes to the development of fatty liver disease. It is, however, suggested that the ensuing hepatic lipotoxicity is potentially driving the development of liver injury and inflammation that is characteristic of NASH (Neuschwander-Tetri, 2010).

### The Role of Hepatic Lipotoxicity

Many patients with fatty liver disease show only steatosis for many years without additional characteristics of NASH (Calzadilla Bertot and Adams, 2016). Although the exact mechanism that drives the development of NASH from simple steatosis is unclear, lipotoxic lipid-inflicted cell injury is proposed to be a major contributor (Neuschwander-Tetri, 2010). In agreement with this hypothesis, common lipotoxic lipids such as cholesterol, TG and DAG are reported to be significantly elevated in the liver of NASH patients when compared to control subjects (Puri et al., 2007; Magkos et al., 2012). Animal studies, where rodents were fed a high fat or high cholesterol diet revealed that liver resident macrophages can be activated by engulfing cholesterol crystals resulting in liver inflammation (Van Rooyen et al., 2011; Mridha et al., 2017). DAG is known for its ability to exacerbate hepatic insulin resistance by interfering with insulin signaling via PKC activation (Samuel et al., 2004; Mota et al., 2016). Moreover, the accumulation of TG in the liver leads to steatosis, which is a hallmark of fatty liver disease (Thomas et al., 2005). The role of other FFA-derived lipid species such as ceramide is inconclusive. NAFLD animal models showed elevated levels of ceramide and inhibition of ceramide synthesis attenuated liver inflammation (Jiang et al., 2019; Montandon et al., 2019). Nevertheless, clinical observations from Magkos et al. (2012) reported that the severity of NAFLD/NASH is not correlated with hepatic ceramide content, although it is worth noting that this clinical study only included a small patient population of 16 subjects (Magkos et al., 2012). Interestingly, another study reported an elevated ceramide in the adipose tissue of obese and insulin resistant human subjects (Turpin et al., 2014). Clinical studies involving a wider patient cohort is warranted to confirm the findings from preclinical studies.

### The Role of Hepatic Oxidative Stress

Oxidative stress-induced hepatocyte damage and apoptosis have been reported as one of the main drivers of tissue injury in NASH patients (Masarone et al., 2018). As an adaptation to minimize hepatic steatosis, the rate of the disposal of fatty acid via mitochondrial ß-oxidation was reported to be significantly upregulated in NAFLD and NASH patients (Sanyal et al., 2001). However, studies comparing mitochondrial function in NAFLD and NASH patients have highlighted that this adaptation is lost in later stage-NASH patients, due to excessive ROS-induced mitochondrial dysfunction (Kojima et al., 2007; Koliaki et al., 2015). Apart from mitochondria-derived ROS, NADPH oxidase 2 (NOX2) activation in liver-infiltrating macrophages is also reported to contribute to oxidative stress-induced liver damage in NAFLD (Kim et al., 2017). More importantly, Yesilova and colleagues (2005) documented that NAFLD/NASH patients exhibit the reduced activity of antioxidative mechanisms such as coenzyme Q10 and superoxide dismutase. In addition, the reduced glutathione: oxidized glutathione (GSH:GSSG) ratio in animals with diet-induced with NASH also highlights an imbalance between ROS and antioxidants (Iruarrizaga-Lejarreta et al., 2017). Compromised antioxidant capacity enables the generation of reactive oxygen/nitrogen species such as hydroxyl radical (^•^OH), superoxide anion (O_2_^−•^) and peroxynitrite (ONOO^−^) to accumulate and readily react with intracellular biomolecules, such as FFAs and DNA (Fujita et al., 2010; Mello et al., 2016). As a result, by-products of reactive oxygen species (ROS)-induced damage such as 4-hydroxynonenal and 3-nitrotyrosine was significantly enhanced in the plasma and liver, respectively in NAFLD/NASH patients (Loguercio et al., 2001; Kojima et al., 2007).

### The Role of Hepatic ER Stress

Similar to oxidative stress, upregulated hepatic ER stress is closely associated with NASH (Lake et al., 2014). Kuo et al. (2012) provided evidence that an ER stress response is provoked in response to an increase in FFA accumulation in hepa. Consistent with this observation, Xiao et al. (2013) demonstrated that mice deficient in activating transcription factor 4 (ATF4), a major ER-stress mediator, were protected from high fructose diet-induced hepatic steatosis, highlighting the necessity of the ER stress response in driving the accumulation of fat in the liver. In general, when the concentration of intracellular unfolded proteins reaches a critical threshold, the ER initiates the unfolded protein response (UPR) in an attempt to maintain normal cell function (Lebeaupin et al., 2018). The UPR encompasses three main pathways: reduced protein translation by activating protein kinase RNA-like endoplasmic reticulum kinase-eukaryotic initiation factor 2 alpha (PERK-eIF2α) signaling (Harding et al., 1999); enhancing protein folding via the inositol-requiring enzyme 1 (IRE1) and X-box binding protein 1 (XBP1) signaling cascade (Ning et al., 2011), and inducing apoptosis and ER-associated degradation by activating transcription factor 6 (ATF6) associated pathway (Lebeaupin et al., 2018). However, prolonged unresolved ER stress is thought to induce the expression of the pro-apoptotic transcription factor C/EBP Homologous Protein (CHOP) (Zinszner et al., 1998). In an ER-stress induced NASH model induced by major urinary protein urokinase-type plasminogen activator (MUP-uPA, discussed in detail later), animals exhibit high levels of XBP1s as well as CHOP (Nakagawa et al., 2014). Although apoptosis was elevated together with increased CHOP expression, liver injury was not ameliorated in mice with CHOP ablation (Soon et al., 2010; Nakagawa et al., 2014). It is possible that CHOP is a downstream product of ER stress but not an active driver of liver injury in NAFLD/NASH. Clinical studies displayed varying degrees of ER stress gene and protein expression in NASH patients (Puri et al., 2008; Lake et al., 2014). It is noteworthy that both studies showed high variability within the NASH patient cohort, with Puri et al. (2008) having 21 NASH patients and Lake et al. (2014) having 13 NASH patients. Given the complexity of NAFLD/NASH pathology, the expression levels of ER stress mediators may be influenced by many different factors. The different results observed in these studies may be attributed to patient variability. Recruitment of a larger patient cohort and effective patient stratification may provide a better understanding of the drivers underpinning ER-stress-driven liver injury.

### The Role of c-Jun N-Terminal Kinase Signaling

There are numerous *in vitro* and *in vivo* studies highlighting the pleiotropic role of intracellular signaling pathways such as JNK in the development of NASH (Gehrke and Schattenberg, 2020). In particular, JNK activation by TNF-α has been implicated in mediating insulin resistance by interfering with the IRS signaling pathway (Aguirre et al., 2000). In addition, FFA-induced JNK activation resulted in cell apoptosis in both cell line cells and primary mouse hepatocytes (Malhi et al., 2006). In contrast, hepatocytes that are isolated from JNK1 deficient mice had reduced apoptosis compared to cells from wild type animals when exposed to FFAs (Malhi et al., 2006). In support of this finding, mice genetically deficient in JNK1 exhibit attenuated hepatic steatosis compared to their wild type counterparts in a dietary model of NASH (Schattenberg et al., 2006). Interestingly, it has been reported that JNK1 deficiency in the adipose tissue indeed protects animals against hepatic steatosis (Sabio et al., 2009). However, JNK1 deficiency in the liver gave rise to glucose intolerance and insulin resistance in these animals with diet-induced NASH (Sabio et al., 2009). Therefore, future therapeutic targeting of the JNK pathway may need to take into consideration the differential effects that JNK1 blockade might have at different target organs.

### The Role of Hepatic Inflammation

Inflammation is one of the features that distinguishes NASH from fatty liver disease (Kleiner et al., 2005). Although the exact mechanism that triggers inflammation in NASH patients is not well characterized, several key contributing factors have been suggested (Kleiner et al., 2005; Younossi et al., 2011). Adipose tissue-derived cytokines, such as TNF-α, are suggested to contribute to hepatic inflammation (Hotamisligil et al., 1993; Tilg and Moschen, 2006). In addition, gut dysbiosis caused by long-term HFD consumption can result in a leaky gut, enabling endotoxins, such as lipopolysaccharide to travel to the liver, triggering/enhancing liver inflammation during NASH (Hildebrandt et al., 2009; Ogawa et al., 2018). Moreover, metabolism associated molecular patterns (MAMPS) including FFAs and cholesterol have been reported to initiate inflammasome-induced inflammatory cell death in hepatocytes (Csak et al., 2011; Mridha et al., 2017; Wang et al., 2020). The resulting danger-associated molecular patterns (DAMPs) from inflammatory cell death can stimulate the activation of liver resident macrophages known as Kupffer cells (Seki and Brenner, 2008; Baffy, 2009). Activated Kupffer cells secrete TNF-α (Tosello-Trampont et al., 2012), a pro-inflammatory cytokine that mediates pleiotropic actions including amplifying insulin resistance and regulating NF-κB activation (Schütze et al., 1995; Peraldi et al., 1996). NF-κB has been suggested as a key player in exacerbating liver inflammation, as phosphorylated NF-κB levels are also elevated in preclinical models of NASH (dela Peña et al., 2005; Nakagawa et al., 2014). Moreover, pharmacological inhibition of NF-κB activation significantly reduced the expression of NF-κB downstream inflammatory genes in animal models of NASH (Leclercq et al., 2004). In addition to NF-κB activation, TNF-α also induces the expression of monocyte chemoattractant protein-1 (MCP-1) which is reported to be elevated in NASH patients (Haukeland et al., 2006; Greco et al., 2008; Tosello-Trampont et al., 2012). MCP-1 and its corresponding receptor, C-C chemokines receptor type 2 (CCR2), are important for the hepatic recruitment of Ly6C^+^ monocytes, which can amplify inflammation as they mature into macrophages (Baeck et al., 2012; Miura et al., 2012). In addition to monocytes and Kupffer cells, neutrophil-secreted myeloperoxidase has been proposed to exacerbate liver inflammation by generating oxidative stress (Rensen et al., 2009). In agreement with these findings, Zang and colleagues (2015) discovered that neutrophils are responsible for contributing to liver inflammation during early stages of NASH. Animals with Ly6G^+^-neutrophil depletion in the early stages of NASH displayed significantly reduced serum ALT, as well as reduced pro-inflammatory gene expression compared to diseased mice (Zang et al., 2015). Recent studies demonstrate that patients who progress to NASH exhibit a high level of natural killer T-cells and CD8^+^-T-cells (Tajiri et al., 2009; Gadd et al., 2014). A potential role for T-helper cells in mediating NASH progression and initiation of fibrogenesis has indeed been proposed (Rolla et al., 2016).

### The Role of Hepatic Fibrosis

Hepatic fibrosis one of the hallmarks of NASH, is characterized by extensive accumulation of connective tissue which following extensive tissue damage (Kleiner et al., 2005). The process of fibrogenesis in the liver is thought to be mainly regulated by hepatic stellate cells (HSCs), a type of liver progenitor cell that is quiescent under physiological conditions (Tsuchida and Friedman, 2017). HSC can be activated to produce collagen I in response to elevated ER stress by overexpressing XBP1 (Kim et al., 2016). Apart from ER stress, liver-specific overexpression of NACHT, Leucine-rich-repeat and pyrin domain-containing protein 3 (NLRP3) induced marked HSC activation and fibrosis, indicating a possible role of inflammatory cell death in inducing HSC activation (Wree et al., 2014). Moreover, it has been elucidated that engulfment of apoptotic cell bodies induces HSC activation (Canbay et al., 2003). The activated HSC transforms from a dormant cell into an active myofibroblast which is characterized by increased production of collagen I, collagen III and transforming growth factor-β (TGF-β) (Dooley et al., 2000; Inagaki et al., 2001; Zhan et al., 2006). More importantly, TGFβ acts in a paracrine/autocrine fashion to activate quiescent HSCs, while also amplifying collagen deposition from activated HSCs (Hellerbrand et al., 1999). The increased collagen I and III gradually alters the composition of liver extracellular matrix and gave rise to tissue scarring (Maher and McGuire, 1990; Mak and Mei, 2017). If liver injury is not resolved, the continuous supply of inflammatory cytokines and apoptotic cell bodies will perpetuate the fibrogenic actions of HSC and promote further tissue remodeling (Bachem et al., 1992). When the collagen deposition is evident in most of the liver tissue, the disease has officially progressed beyond NASH to cirrhosis (Kleiner et al., 2005). Moreover, results from longitudinal studies suggested that NAFLD/NASH patients with severe liver fibrosis have increased risk of HCC and mortality compared to those with mild fibrosis (Ekstedt et al., 2015; Alexander et al., 2019).

## Preclinical Models of Non-Alcoholic Steatohepatitis

NASH is a disease that encompasses a broad array of systemic metabolic disruptions as well as liver-specific abnormalities induced by a multitude of processes (Buzzetti et al., 2016). The complex nature of the disease has made it challenging to recapitulate the full spectrum of the disease phenotype in animal models (Friedman et al., 2018a). The currently established models are broadly categorized into three main areas: dietary-induced, diet-toxin-induced and diet-genetically mutated models (Table 2).

**TABLE 2:** Summary table of commonly used preclinical models of NASH.

Induce method	Models	Weight gain	Insulin resistance	Hepatic steatosis	Hepatic inflammation	Hepatic fibrosis	References
Diet-induced model	MCD	✕	✕	↑↑	↑↑	↑↑	(Rinella and Green, 2004; Rinella et al., 2008)
	CD	✕	✕	↑↑	✕	✕	(Caballero et al., 2010)
	MD	✕	✕	↑	↑↑	↑↑	(Caballero et al., 2010)
	HFD	↑↑	↑↑	↑↑	↑	✕	(Kohli et al., 2010; Mulder et al., 2016)
	WD	↑↑	↑↑	↑↑	↑	✕	(Machado et al., 2015; Schierwagen et al., 2015)
	AMLN*	↑↑	↑↑	↑↑	↑↑	↑	(Clapper et al., 2013; Boland et al., 2019)
Diet + Toxin-induced model	STAM	✕	✕	↑↑	↑↑	↑↑	(Fujii et al., 2013; Orime et al., 2016; Middleton et al., 2018)
	HFD + DEN	✕	✕	↑↑	↑↑	✕	(Kishida et al., 2016)
	WD + CCL_4_	✕	✕	↑↑	↑↑	↑↑	(Baeck et al., 2012; Tsuchida et al., 2018)
Genetic/+diet models	*ob/ob*	↑↑	↑↑	↑↑	↑	✕	(Trak-Smayra et al., 2011; Sutter et al., 2015)
	*db/db*	↑↑	↑↑	↑↑	↑	✕	(Sahai et al., 2004; Trak-Smayra et al., 2011)
	foz/foz*	↑↑	↑↑	↑↑	↑	↑	(Van Rooyen et al., 2011; Haczeyni et al., 2017; Mridha et al., 2017)
	ApoE−/−*	↑	↑	↑	↑	↑	(Schierwagen et al., 2015)
	hApoE-KI*	↑	↑	↑	↑	↑	(Shiri-Sverdlov et al., 2006; Staels et al., 2013)
	MUP-uPA + HFD	↑↑	↑↑	↑↑	↑↑	↑↑	(Nakagawa et al., 2014; Febbraio et al., 2019)
	PNPLA3 I148M	✕	✕	↑	✕	✕	(Smagris et al., 2015)

Summarized key characteristics of NASH. ↑: modest increase; ↑↑: marked increase; ✕: did not exhibit this feature; *: shortcomings of this model mentioned in the text; HFD: High fat diet; WD: Western diet; MCD: methionine choline deficient diet; MD: methionine deficient diet; CD: choline deficient diet; AMLN (Amylin NASH diet): HFD containing cholesterol supplemented by high fructose and sucrose; STAM: streptozotocin + HFD; DEN: diethylnitrosamine; *ob/ob*: leptin-deficient mice; *db/db*: leptin-receptor-deficient mice; *foz/foz*: *Alms1* genetic mutation mice; *ApoE−/−*: Apolipoprotein E Knock out; hApoE-KI: human Apolipoprotein E Knock in; *PNPLA3I148M* KI: 148 Isoleucine to Methionine protein variant (I148M) of patatin-like phospholipase domain-containing three knock-in mice; *MUP-uPA*, mice overexpressing urokinase plasminogen activator introduced into hepatocytes via adeno-associated virus.

### Genetically Induced Non-Alcoholic Steatohepatitis Models

Genetically-induced obese mouse models of diabetes and pre-diabetes, such as *ob/ob*, *db/db* and *foz/foz* exhibit are also being used as models of NASH/NAFLD as they exhibit obesity, insulin resistance and hyperglycaemia (Bleisch et al., 1952; Marshall et al., 2011).

#### *ob/ob* and *db/db* Models

The *ob* gene transcribes leptin, an adipocyte hormone involved in the regulation of food intake and insulin sensitivity (Friedman et al., 1991). In *ob/ob* mice, there is a deficiency in the production of functional leptin (Friedman et al., 1991; Zhang et al., 1994). Therefore, animals with this genetic alteration develop hyperphagia and insulin resistance (Friedman and Halaas, 1998). Sutter and colleagues (2015) demonstrated that *ob/ob* mice fed with an HFD rapidly gained weight and developed insulin resistance and most of the liver NASH features except fibrosis. Complementing this finding, another study by Leclercq et al. (2002) reported that leptin is essential to promote liver fibrosis. Thus, the *ob/ob* model is deemed unsuitable for studying NASH due to this paradoxical shortcoming. Having a similar metabolic phenotype to the *ob/ob* animals, the *db/db* model exhibits leptin resistance caused by premature termination of leptin receptor transcription (Chen et al., 1996). Disruption in transcription gave rise to faulty leptin receptors which precluded normal leptin signaling (Chen et al., 1996). The *db/db* model gave rise to severe obesity, glucose intolerance and liver steatosis. Nonetheless, liver inflammation and fibrosis in this model were reported to be mild (Trak-Smayra et al., 2011). Several studies use *db/db* mice coupled with a methionine-choline-deficient (MCD) diet feeding to induce more severe liver injury (Sahai et al., 2004; Rinella et al., 2008; Staels et al., 2013). More importantly, it has been proposed that whilst *ob/ob* and *db/db* mice can be good models for studying obesity and insulin resistance, both *ob* and *db* mutations are rare in humans, therefore, these mice may be less clinically-relevant as animal models of NASH (Carlsson et al., 1997; Wang et al., 2014).

#### *foz/foz* Model

The *foz/foz* mice have also been used as an obese and diabetic NASH model (Van Rooyen et al., 2011; Haczeyni et al., 2017; Mridha et al., 2017). The *foz/foz* mice were generated from a recessive mutation on the AlstrÖm syndrome 1 (*Alms1*) gene which encodes proteins involved in ciliary function (Marshall et al., 2011). Mice that have genetic mutation typically develop hyperinsulinemia, hyperglycaemia, and hypercholesterolemia together with liver inflammation (Van Rooyen et al., 2011). Nevertheless, obeticholic acid (OCA), an FDA-approved drug for NASH (discussed in detail below), did not improve liver histology of *foz/foz* mice, like it did in NASH patients (Haczeyni et al., 2017; Younossi et al., 2019). This finding raises a question regarding the use of animal models that are merely a “phenocopy” of human NASH, as humans do not normally develop NASH due to the rare autosomal recessive *Alms1* mutation (Marshall et al., 2011).

#### Apolipoprotein E Knock-Out and Knock-In Models

ApoE is a multifunctional protein that plays an important role in lipid transport, abnormality in the type 2 ApoE results in type III hyperlipoproteinemia (Huang and Mahley, 2014). Mice that are ApoE deficient (ApoE−/−) are commonly used as an animal model for atherosclerosis (Song et al., 2011). Although weight gain and abnormal glucose tolerance can be achieved in ApoE−/− animals, the model by itself only gave rise to negligible hepatic steatosis, inflammation and fibrosis (Schierwagen et al., 2015). Only when ApoE deficiency is combined with a high caloric diet, or a MCD diet does it then induce extensive liver injury (Schierwagen et al., 2015; Zang et al., 2015). Furthermore, hApoE2-knock-in mice, where the human ApoE2 gene replaced the murine gene, is used as another model for NASH studies (Shiri-Sverdlov et al., 2006; Staels et al., 2013). Interestingly, despite inflammatory and fibrotic genes both being upregulated in the liver, only mild steatosis was observed in the hApoE2 mice (Shiri-Sverdlov et al., 2006). Noteworthily, there are few clinical studies examining the association between ApoE2 polymorphism and NAFLD. In one clinical study 57 NAFLD patients from a Turkish ethnic background showed no significant association between ApoE2 and NAFLD (Sazci et al., 2008). Results from this study are consistent with a case controlled study by Demirag et al. (2007) involving 237 NAFLD patients, where subjects with ApoE2 polymorphism showed a significant association with dyslipidaemia but not with NAFLD. The ability to generate a model with dyslipidaemia was what made ApoE−/− and hApoE2 KI mice a potential model for the study of NAFLD/NASH (Sazci et al., 2008). However, Severson et al. (2016) in their systematic clinical review concluded that ApoE polymorphism may not play as important a role as other genetic polymorphisms such as PNPLA3.

#### Phospholipase Domain-Containing-3 Variant-Knock-In Model

Recently, there have been attempts to develop a fatty liver disease mouse model by introducing the human *PNPLA3* polymorphism in mice to mimic human genetic mutant-induced NASH (Smagris et al., 2015). However, mice with human *PNPLA3* variant knock-in (KI) only showed elevated hepatic fat when fed a HFD, whereas the extent of liver inflammation and fibrosis in *PNPLA3* variant KI mice was comparable to wildtype animals (Smagris et al., 2015). It has been suggested that this model may be suitable for the study of fatty liver disease and hepatic insulin resistance (Kumashiro et al., 2013). Further studies of the *PNPLA3* polymorphism in the context of NASH in humans and its mechanism of action are required, to confirm whether murine models with this genetic mutation are good preclinical models of NASH.

#### Major Urinary Protein Urokinase-Type Plasminogen Activator Model

A relatively new NASH model has been developed by transiently upregulating ER stress in the liver by delivering major urinary protein urokinase plasminogen activator (*MUP-uPA*) into the hepatocytes via adeno associated virus coupled with a HFD (Nakagawa et al., 2014; Febbraio et al., 2019). The method of generating *MUP-uPA*-transgenic mice was first described by Weglarz et al. (2000). The *MUP-uPA* transgenic mice is generated by delivering adeno-associated virus containing the uPA protein specifically to hepatocytes. This results in an accumulation of uPA protein in the ER of hepatocytes and thus transiently upregulates ER stress in the hepatocytes (Nakagawa et al., 2014). The *MUP-uPA* mice placed on a HFD exhibited significantly upregulated liver injury markers of NASH, including ER-stress, fibrosis and inflammation at 24 weeks (Nakagawa et al., 2014; Lebeaupin et al., 2018). Furthermore, *MUP-uPA* mice spontaneously progress from NASH to HCC by 32 weeks of age, exhibiting markers frequently observed in humans HCC tissues such as alpha fetoprotein and p62 (Nakagawa et al., 2014). In support of this finding, Shalapour et al. (2017) observed an elevation of immunosuppressive IgA^+^ cells, interleukin 10 and programmed cell death ligand-1 in both NASH-derived HCC patients and the *MUP-uPA* mice that were placed on HFD. More importantly, some degree of transcriptomic alignments were observed between human NASH/HCC subjects and the *MUP-uPA* model, highlighting the clinical relevance of this model (Febbraio et al., 2019).

### Diet-Induced Models

Other than genetic predisposition, a diet high in fat and sugar is one of the major factors that is strongly associated with the development of NASH in humans (Faeh et al., 2005; Luukkonen et al., 2018). Diet-induced NASH models include, but are not limited to, MCD diet (Rinella et al., 2008), HFD (HFD) (Kohli et al., 2010), western diet (WD) (Bruckbauer et al., 2016) and Amylin diet (AMLN) (Clapper et al., 2013; Asgharpour et al., 2016) with only the most widely used models summarized here. MCD is a dietary model used for inducing NASH-like liver features with 40% of sucrose and 10% energy derived from lipids but is deficient in methionine and choline (Anstee and Goldin, 2006). Methionine and choline are essential nutrients for growth and development in humans (Zeisel and Da Costa, 2009). Feeding a diet which is deficient of these two nutrients can lead to the rapid development of hepatic lesions such as hepatic steatosis, inflammation and fibrosis (Oz et al., 2008). Nevertheless, Rinella et al. (2004) showed significantly lower body weight and unaltered plasma insulin in MCD diet-fed mice, highlighting the absence of key metabolic characteristics of NASH such as insulin resistance and obesity in this model. Overall, the field has reached a consensus that the MCD can exhibit histological features that are not only similar to, but are equally severe, as those in human NASH, though key metabolic features are missing (Leclercq et al., 2000; Rinella and Green, 2004; Rinella et al., 2008; Wortham et al., 2008). There have been attempts to use only methionine-deficient (MD) or choline deficient (CD) diets to induce NASH (Caballero et al., 2010). Despite animals on MD and CD had reduced weight loss, metabolic features that are present in NASH in human was not observed (Caballero et al., 2010; Febbraio et al., 2019). To develop a NASH model that mimics both systemic and hepatic pathology, many attempts have been made using varying degrees of fat (∼40–70% energy derived from fat) and 0.1–2% cholesterol in the diet (Anstee and Goldin, 2006). The use of high-fat content alone is normally referred to as the HFD model (Kohli et al., 2010), whereas WD represents a type of HFD with the addition of cholesterol (Machado et al., 2015; Bruckbauer et al., 2016). Models that received HFD or WD feeding develop weight gain, insulin resistance and hepatic steatosis which are concordant with the insulin resistance and hyperglycaemia of humans who have NASH (Zheng et al., 2008; Kohli et al., 2010). However, in some cases, HFD and WD models are reported to have minimal fibrosis (Febbraio et al., 2019). In recent years, the ALMN diet-induced NASH model developed by Amylin Pharmaceuticals (hence ALMN model), composed of 40% lipids, 2% cholesterol and water supplemented with fructose, has been reported to display both systemic and liver-specific characteristics of human NASH at 28–30 weeks of AMLN diet feeding (Clapper et al., 2013; Boland et al., 2019). Overall, NASH animal models induced by dietary interventions alone require a long time to achieve a mild to moderate NASH phenotype. Characteristics such as moderate to severe liver injury and fibrosis may take up to 20–30 weeks of dietary feeding (Charlton et al., 2011; Clapper et al., 2013). However, the longer the study period of animal experiments, the higher the chance of animals dying due to aging and age-related complications. In addition, such models are resource-draining and less time-effective. Thus, other alternative models are being explored in the attempt to induce severe liver injury in a shorter period.

### Diet and Toxin-Induced Models

To increase the severity of liver injury in rodent NASH models, toxins such as streptozotocin (STZ) (Fujii et al., 2013), diethylnitrosamine (DEN) (Park et al., 2010) and carbon tetrachloride (CCL_4_) (Tsuchida et al., 2018) have been added to the modified diet.

#### STAM Model

In the STAM model, a single dose (200 μg) of the pancreatic ß-cell toxin STZ is administered subcutaneously to 2 day old neonatal C57BL/6 mice followed by 4–6 weeks of HFD feeding (Fujii et al., 2013; Saito et al., 2017; Middleton et al., 2018). By destroying pancreatic ß-cells, the STZ-induced hyperglycaemia is coupled with HFD to drive liver damage feeding (Fujii et al., 2013; Saito et al., 2017; Middleton et al., 2018). Although the STAM model gives rise to liver steatosis, inflammation and fibrosis, these animals develop conditions that resemble type 1 rather than type 2 diabetes, as indicated by the overt hyperglycaemia (blood glucose 600 mg/dl) and a lack of hyperinsulinemia, a sign of insulin resistance (plasma insulin <0.5 ng/ml) (Saito et al., 2017). Although the STAM model has been discussed in other reviews, consideration of the combination of STZ and HFD (STZ + HFD) where STZ was delivered at a later stage of the animal’s life is less frequently noted (Friedman et al., 2018a; Farrell et al., 2019; Oligschlaeger and Shiri-Sverdlov, 2020). FVB/N mice which received STZ (55 mg/kg) at 6 weeks old coupled to a HFD displayed hyperinsulinemia (Tate et al., 2019). Moreover, rats fed with a HFD before receiving STZ injection also showed hyperinsulinemia (Reed et al., 2000). The contradictory findings in the literature may be partially explained by variable susceptibility toward STZ in different mouse strains (FVB/N Vs C57BL/6) (Saito et al., 2017; Tate et al., 2019; Marshall et al., 2020). Noteworthily, the STZ model has also been criticized for its ability to damage other organs such as the kidney and the liver via DNA alkylation (Lenzen, 2008). STZ-induced liver injury is thought to be direct rather than secondary to the natural course of NASH-induced liver injury (driven by T2D and obesity) which is one of the major issues limiting the utility of the STAM model (Middleton et al., 2018; Farrell et al., 2019).

#### Diethylnitrosamine + HFD Model

The hepatic carcinogen DEN has been shown to induce severe hepatic injury, by inducing mutagenic DNA damage and upregulating ROS production (Williams et al., 1996). After receiving 25–30 mg/kg of DEN at 14 days old, rodents which were fed a HFD for 4–6 weeks were reported to display severe liver injury characterized by elevated inflammatory gene expression and hepatocyte ballooning (Wang et al., 2009; Park et al., 2010). An important caveat of this model is that DEN + HFD-treated animals rapidly develop hepatocellular carcinoma (HCC) due to the potential carcinogenic effects of DEN (Wang et al., 2009).

#### Western Diet + Carbon Tetrachloride Model

Another NASH model is induced by the hepatotoxin CCL_4_ which rapidly causes severe liver inflammation and fibrosis (Hellerbrand et al., 1999; Baeck et al., 2012). The use of CCL_4_ coupled with a WD is also reported to give rise to weight gain and severe liver histological features similar to those of NASH patients (Tsuchida et al., 2018). Although this model is capable of inducing stage 3 fibrosis after 12 weeks of HFD feeding and CCL_4_ treatment, CCL_4_ induces severe liver injury via oxidative DNA damage, which is distinctly different from the natural course of NASH (Alkreathy et al., 2014; Calzadilla Bertot and Adams, 2016).

NASH is a heterogenous disease characterized by both liver injury and systemic metabolic disruptions (Friedman et al., 2018a). Currently, although diet-induced models such as HFD and ALMN diet-induced NASH models are time consuming and only show mild liver injuries, they recapitulate the natural course of NASH development in humans (Friedman et al., 2018a). The MUP-uPA model, although not widely-used at the moment, does mimic aspects of NASH in humans as oppose to the MCD diet and some toxin-induced NASH models (Febbraio et al., 2019). A summary of all the frequently used animal models of NASH is provided in Table 2.

## Current Interventions in Non-Alcoholic Steatohepatitis Management

### Lifestyle Modification

Management of fatty liver diseases has been addressed by lifestyle modifications, including regular physical exercise and consuming a hypocaloric diet (Vilar-Gomez et al., 2015). Often, a reduction of ≥5–10% of the subject’s body weight is required to achieve attenuation of NASH (Vilar-Gomez et al., 2015; Younossi et al., 2018). Nevertheless, a study also noted a lack of patient compliance with the proposed exercise and nutritional recommendations after the study period (Eckard et al., 2013). More importantly, lifestyle changes alone are insufficient to stop disease progression, especially for patients who are at later stages of the disease where there are ongoing liver inflammation and fibrosis (Promrat et al., 2010). Patients with progressed fibrosis have an increased risk of developing cirrhosis and liver failure, and it is currently the second leading cause of liver transplant (Wong et al., 2015).

### Pharmacological Treatments

Apart from lifestyle modifications, obeticholic acid (OCA), originally approved for the treatment of primary biliary cholangitis (PBC), is the only FDA-approved treatment for NASH (Vilar-Gomez et al., 2015; Younossi et al., 2019). OCA is a farnesoid X receptor (FXR) agonist which regulates the expression of transcription factors that reduce bile acid synthesis and hepatic steatosis (Pellicciari et al., 2002; Jiao et al., 2015). In the FLINT trial (NCT01265498), OCA has been shown to improve liver inflammation with no worsening of liver fibrosis (Neuschwander-Tetri et al., 2015). In the recent 18 months phase III clinical trial REGENERATE (NCT02548351), 23% (71/308) of the patient cohort who received 25 mg daily achieved reduction of NAS by at least one score without worsening of fibrosis compared to 12% (37/311) in the placebo group (Eslam et al., 2019). The trial results enabled OCA to be granted accelerated approval from the FDA (Younossi et al., 2019). Although, there were 19 deaths observed in PBC patients who received obeticholic acid due to incorrect dosing (Eslam et al., 2019). Within in the 19 cases of death, 8 cases were reported. The cause of death for seven patients were due to the worsening of PBC, and the other patient due to cardiovascular complications (FDA website). A safety warning has been issued by the FDA for patients and health professionals regarding the use of obeticholic acid for its potential effect of worsening liver disease in patients.

Vitamin E is an anti-oxidant which acts by reducing the ROS and inflammation-induced liver damage (Singh et al., 2005). Results from a 96 weeks multicenter, placebo-controlled trial showed improvements of liver histology such as inflammation, steatosis and ballooning in 43% (34/84) of the non-diabetic NAFLD subjects treated with 800IU vitamin E daily compared to 19% (16/83) treated with placebo (Sanyal et al., 2010). However, concerns that long-term vitamin E use may be associated with hemorrhagic stroke are also highlighted in the study (Sanyal et al., 2010). Alarmingly, in a separate study where vitamin E (400IU/d) was administered for 7–11 years showed increased risk of prostate cancer was identified in healthy men with long-term vitamin E treatment (Klein et al., 2011).

Pioglitazone primarily targets the PPARγ receptor which ameliorates insulin resistance, an independent predictor of NASH (Belfort et al., 2006). In a 96 weeks placebo-controlled trial, 30 mg of pioglitazone daily also improved the liver histology in 34% (27/80) of non-diabetic NASH patients, although an average of 4.7 kg weight gain was reported in the treatment group (Sanyal et al., 2010; Chalasani et al., 2012). Whilst a recent 18 months study showed that pioglitazone treatment combined with a hypocaloric diet (500 kcal/day deficit) improved liver histology in 51% (26/50) of patients with NASH and diabetes, its efficacy still needs to be evaluated in larger patient cohorts (Cusi et al., 2016).

Currently, both pioglitazone and vitamin E are currently prescribed in a case-by-case manner, as the risk of all-causes of liver-related mortality of these two drugs still need to be evaluated in a larger patient cohort (Sanyal et al., 2010; Chalasani et al., 2012; Younossi et al., 2018). A summary of NASH/NAFLD-related drug treatment is displayed in Table 3.

**TABLE 3:** Summary table of drugs for NASH treatment.

Drug name	Target	Mechanism of action	Trial phase NCT no	Trial population	Outcome (estimated completion date)	References
Aramchol	SCD1 inhibitor	↓DNL synthesis	Phase III NCT04104321	NASH	TBD	FDA website
					June 2022	
		↓Steatosis	Phase II NCT01094158	NAFLD/NASH/MS	Complete	(Safadi et al., 2014)
					Reduced the percentage of liver fat at	
Cenicriviroc	CCR2/5 dual inhibitor	↓Inflammation	Phase III NCT03028740	NAFLD/NASH/MS	TBD	FDA website
					October 2021	
			Phase II NCT02217475	NAFLD/NASH/MS	Complete	(Friedman et al., 2018b)
					Reduced fibrosis with no worsening of inflammation and steatosis	
Elafibranor	PPARα/δ agonist	↓Inflammation	Phase II,I NCT02704403	NAFLD/NASH/MS	Complete (unsuccessful)	(GENFIT S.A, 2020)
					High placebo effect, no difference between placebo arm and treatment arm	
		↓Fibrosis	Phase IIb, NCT01694849	NAFLD/NASH/MS	Complete	(Ratziu et al., 2016)
					Well tolerated in patients. No significant change between placebo and treatment	
		↓Insulin resistance	Phase II, NCT01275469	Obese/pre-diabetic	Complete	(Cariou et al., 2011)
					Improved insulin resistance, decreased fasting TG and blood glucose. Increased HDL	
Emricasan	Pan-caspase inhibitor	↓Inflammation	Phase II NCT02686762	NAFLD/NASH/MS	Complete (unsuccessful)	(Ratziu et al., 2012; Harrison et al., 2020a)
					No improvement in liver histology in patients with NASH, and may exhibit worsened fibrosis and ballooning	
GR-MD-02	Galectin-3 inhibitor	↓Fibrosis	Phase II NCT02462967	NASH, cirrhosis, and portal hypertension	Complete (unsuccessful)	(Chalasani et al., 2020)
					No improvement in hepatic venous pressure and liver histology compared to placebo	
Liraglutide	GLP-1 agonist	↓Insulin resistance	Phase II NCT01237119	NASH/NAFLD/MS	Complete (Preparing for phase III)	(Armstrong et al., 2016)
		↓Blood glucose			Reduced body weight and liver steatosis, and plasma ALT/AST	
		↓Body weight				
Semaglutide	GLP-1 agonist	↓Insulin resistance	Phase II NCT02453711	Obese/Type 2 diabetic	Complete (successful, not for NASH)	(Newsome et al., 2019)
		↓Blood glucose			Reduced plasma ALT and significant weight loss	
		↓Body weight				
Obeticholic acid	FXR agonist	↓Bile acid synthesis	FDA-approved PHASEIII NCT02548351	NASH/NAFLD/MS	Complete (successful)	(Eslam et al., 2019)
		↓Inflammation			23% patients in the 25 mg group had improved fibrosis, but had increased pruritus	
		↓Fibrosis				
Selonsertib	ASK1 inhibitor	↓Cell apoptosis	Phase III NCT03053050 NCT03053063	NASH/NAFLD/MS	Complete (unsuccessful)	(Harrison et al., 2020b)
		↓Inflammation			Neither trial improved fibrosis without worsening of NASH	
		↓Fibrosis				
Pioglitazone	PPARγ agonist	↓Insulin resistance	NCT00063622 Phase III	Non-diabetic NAFLD	Completed (successful)	(Sanyal et al., 2010)
					Lowered plasma ALT/AST, liver inflammation and steatosis	
Vitamin E	Antioxidant	↓Oxidative stress	NCT00063622 Phase III	Non-diabetic NAFLD	Completed (successful)	(Sanyal et al., 2010)
					Lowered plasma ALT/AST, liver inflammation and steatosis	

MS: metabolic syndrome; TG: triglyceride; HDL: high-density lipoprotein; SCD1: stearoyl CoA desaturase 1, CCR2/5: C-C chemokines receptor type 2/5, GLP-1: glucagon-like peptide-1, FXR: Farnesoid X receptor, ASK1: Apoptosis signal-regulating kinase 1, PPARγ: peroxisome proliferator-activated receptor γ.

### Pharmacological Treatments Under Clinical Trial

**FIGURE 3:**
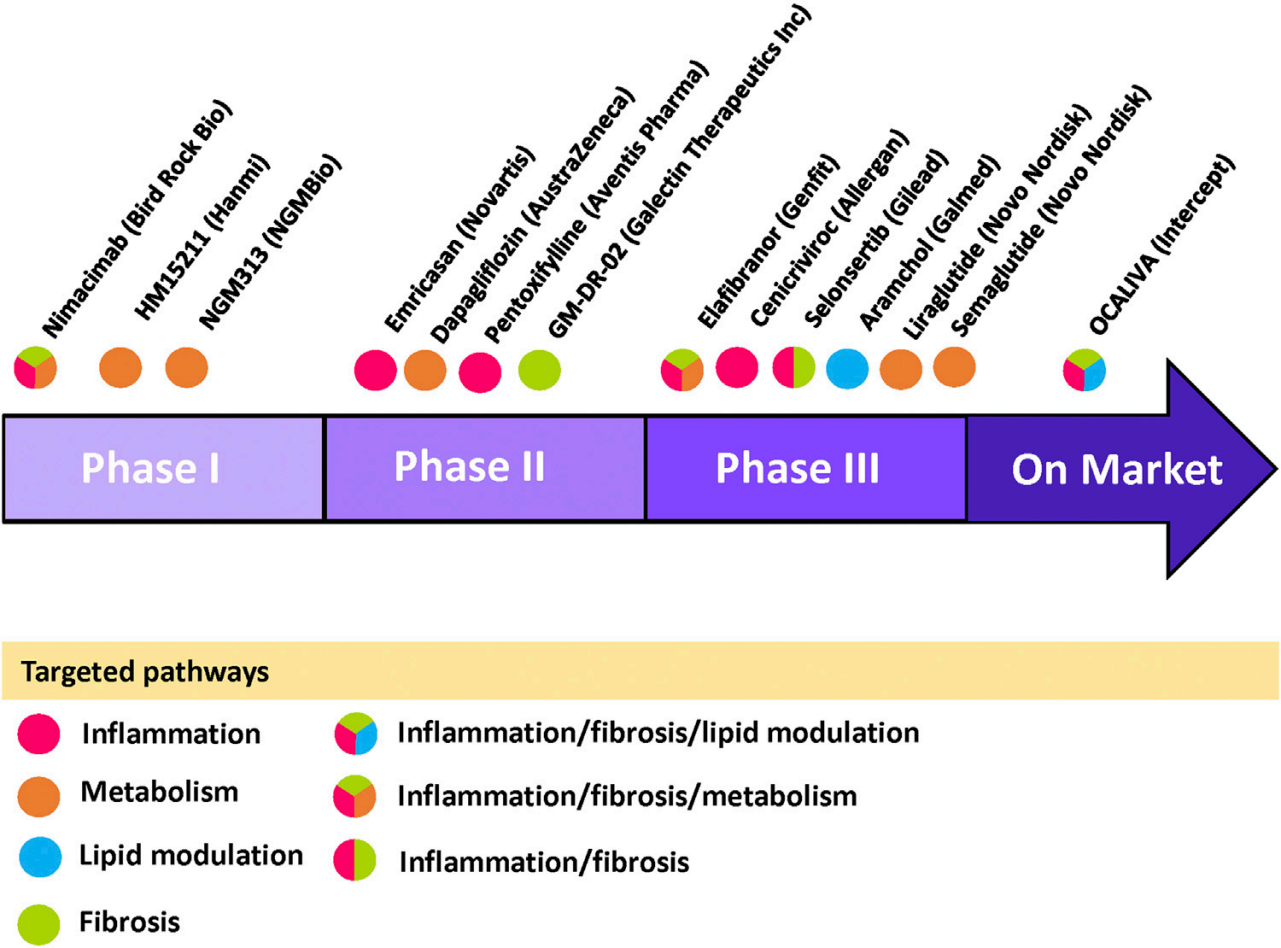
Current NASH/NAFLD pipeline drugs with targeted pathways Pipeline drugs labeled with its pharmaceutical company are placed in their corresponding trial phases. The circle color indicates its targeted pathway(s) as shown in the legend within the figure. Information are gathered from clinicaltrials.gov and pharmaceutical company websites. Figure is designed and drawn using Microsoft Powerpoint.

Most phase IIb and phase III clinical trials of NASH generally have two primary clinical endpoints: 1) resolution of NASH without worsening of liver fibrosis and/or 2) improving liver fibrosis without worsening of NASH (clinicaltrials.gov). Resolution of NASH generally refers to a reduction of NAS, whereas improvement of liver fibrosis refers to reduction in fibrosis scores by liver histology (Kleiner et al., 2005). Many pharmacological treatments are undergoing clinical trials (Smeuninx et al., 2020). Drug candidates from their corresponding pharmaceutical companies and the pathways they are targeting are presented in Figure 3.

#### Glucagon-Like Peptide 1 Receptor Agonists

Synthetic long-acting glucagon-like peptide 1 (GLP-1) receptor agonists such as liraglutide and semaglutide were originally approved for treatment of type 2 diabetes (Pearson et al., 2019). Recently, both liraglutide and semaglutide have gained attention for their efficacy in attenuating insulin resistance, hyperglycemia and liver lipotoxicity in NASH patients (Armstrong et al., 2013; Armstrong et al., 2016). GLP-1, a hormone secreted by the small intestine after a meal, has been observed to restore insulin sensitivity and attenuate hyperglycemia in humans (Garber et al., 2009). Treatment of NASH with GLP-1 receptor agonists was reported to ameliorate liver steatosis in both preclinical and clinical studies (Ding et al., 2006; Armstrong et al., 2013). Novo Nordisk has completed its 48 weeks phase II clinical trial (NCT02970942) assessing the efficacy of 1.8 mg liraglutide given daily and it is preparing for its phase III clinical trial. Moreover, semaglutide, a structurally-related analogue of GLP-1 receptor agonist, significantly reduced body weight and liver enzymes in obese and T2D patients (Newsome et al., 2019). Information from a 72 weeks multicenter phase II trial for semaglutide (NCT02970942) showed that, 33 of 56 NASH patients who received 0.4 mg semaglutide had NASH resolution compared to 10 of 58 patients on placebo (Newsome et al., 2020). Semaglutide was well tolerated with the reported adverse event being gastrointestinal events (Newsome et al., 2020).

#### DNL Enzyme Inhibitors

*De novo* lipogenesis pathway enzymes are another popular target for pipeline drugs. Aramchol, is a synthetic molecule created by conjugating bile acid and arachidic acid (Safadi et al., 2014). Aramchol acts by inhibiting the SCD-1 enzyme, which is a key rate limiting enzyme that is responsible for converting FA into TG (Softic et al., 2016). Aramchol has displayed antioxidative, and anti-fibrotic effects in animal studies whilst reducing hepatic steatosis (Iruarrizaga-Lejarreta et al., 2017). A phase II clinical trial for (NCT01094158) showed that NASH patients treated with 300 mg aramchol daily had liver fat reduced by 12.6–22.1% as compared to the placebo group in which case the liver fat increased by 6.4–36.3% (Safadi et al., 2014). Aramchol is currently undergoing phase III trial (NCT04104321) with an estimated completion date of June 2022.

#### Anti-Inflammatory and Anti-Apoptotic Drugs

Liver inflammation, one of the hallmarks of NASH is also one of the popular targets of pipeline drugs. Several agents targeting inflammation, such as emricasan, a pan caspase inhibitor were observed to be unsuccessful in meeting the primary clinical trial endpoints (Harrison et al., 2020a). Similarly, apoptosis signal-regulating kinase 1 (ASK1) inhibitor selonsertib which acts to prevent hepatocyte apoptosis, displayed promising results in reversing fibrosis and lowering liver inflammation in various preclinical models (Alexander et al., 2019; Challa et al., 2019). However, selonsertib did not reach its primary clinical endpoint, i.e., reversing fibrosis, in either of its phase III trials (STELLAR3: NCT03053050, STELLAR4: NCT03053063). It is worth noting that animal models have limited life-span compared to humans. It is difficulty to accurately determine whether the treatment in animal models is reducing fibrosis or merely delaying its progression.

Cenicriviroc, a CCR2/CCR5 dual-inhibitor is currently undergoing phase III trial with an estimated completion date around October 2021 (NCT03028740). CCR2 is one of the major mechanisms for the recruitment of extrahepatic inflammatory cells (Karlmark et al., 2009; Miura et al., 2012). Inhibition of CCR2 has been shown to exhibit anti-inflammatory effects in the liver in animal studies (Baeck et al., 2012; Krenkel et al., 2018). More importantly, 20% (23/145) of patients receiving 150 mg of cenicriviroc daily had reduced fibrosis as opposed to 10% (14/144) of subjects receiving placebo in its phase II clinical trial (Friedman et al., 2018b; Lefere et al., 2020). Overall, the level of inflammation was reduced in patients receiving cenicriviroc compared to controls (Friedman et al., 2018b; Lefere et al., 2020).

#### PPAR Agonist

Elafibranor, a peroxisome proliferator-activated receptor α/δ (PPARα/δ) dual agonist, was one of the drugs that demonstrated efficacy in improving NASH histology in its phase II trial with 274 patients (Ratziu et al., 2016). Preclinical models used for validation of elafibranor include *db/db* mice, CCL_4_-induced liver fibrosis model and hApoE2-KI mice coupled to WD (Staels et al., 2013). PPARα activation improves NASH by increasing FFA ß-oxidation (Stienstra et al., 2007) and lowering inflammation via negative cross-talk with NF-κB (Delerive et al., 1999). PPARδ is responsible for improving hepatic and systemic insulin sensitivity (Lee et al., 2006). Elafibranor attenuated fibrosis in the CCL_4_-induced liver fibrosis model (Staels et al., 2013; Tsuchida et al., 2018). Moreover, elevated TG, VLDL and inflammatory gene expression exhibited by the WD-fed hApoE2-KI model were also normalized by elafibranor (Shiri-Sverdlov et al., 2006; Staels et al., 2013). However, neither of the CCL_4_ and the WD + hApoE2-KI models exhibit obesity or hyperglycemia (Shiri-Sverdlov et al., 2006; Tsuchida et al., 2018). Elafibranor’s efficacy in improving glucose homeostasis and insulin sensitivity was separately demonstrated in obese *db/db* mice (Hanf et al., 2014). Nevertheless, elafibranor did not achieve its primary clinical endpoint in its recently completed 72 weeks phase III trial (RESOLVE-IT: NCT02704403). Results and interim analysis of the RESOLVE-IT trial showed no significant difference between the placebo arm and the treatment arm (120 mg/daily) (GENFIT S.A, 2020). Although, the full dataset will not be released until the second half of 2020 at an international hepatology congress (GENFIT S.A, 2020). While many reasons may have contributed to the failure for candidate drugs to successfully move from pre-clinical studies to the clinic, the use of animal models that are only partially mimicking the NASH phenotype (as highlighted in the models’ section) may be an important factor. Nevertheless, the full dataset from the phase III clinical study of elafibranor will not be released until the second half of 2020 at an international hepatology congress (GENFIT S.A, 2020). Further analysis of the existing clinical data is required to determine the therapeutic effect of long-term elafibranor treatment in a large trial population.

#### Plant-Based Natural Products

In recent years, there has been growing interests in using plant-based natural products or extracts for the treatment of NASH (Jadeja et al., 2014). Many of these products are widely-used as traditional Chinese medicine and are now being investigated for their potential beneficial effect for NASH in preclinical models (Jadeja et al., 2014; Sun et al., 2017). Plants including *Acanthopanax senticosus* (Siberian Ginseng) (Park et al., 2006) and glycyrrhizic acid (Sun et al., 2017) showed reduced hepatic *de novo* lipogenesis and improved insulin sensitivity in mouse models of NASH. Likewise, a series of natural-product-derived analogues are also being tested for therapeutic potential in mice with diet-induced NASH and have been shown to lower hepatic lipogenesis as well as ER stress and oxidative stress (Rao et al., 2015; Rao et al., 2019; Rao et al., 2020). Moreover, the use of herbal medicine for the induction of autophagy as a treatment for NASH/NAFLD has been thoroughly reviewed by Zhang et al. (2018). Nevertheless, large-scale clinical trials involving participants from multiple ethnic background are required to confirm the therapeutic potential of plant-based natural products for counteracting NASH.

## Perspectives

In order to further bridge the gap between preclinical and clinical studies, animal studies should exploit publicly-available gene profiling data derived from biopsies of healthy controls and NASH patients to verify the animal models (Morrison et al., 2018). The “multiple-omits” approach, incorporating proteomics and lipidomics into the preclinical studies may give an integrated understanding of the animal model and can better assess its translatability as a preclinical model for human NASH (Hasin et al., 2017). Currently, proteomics and lipidomic analyses have often been used for the identification of potential non-invasive biomarkers of NASH in humans, which can also potentially shed light on patient stratification (Puri et al., 2007; Puri et al., 2009; Niu et al., 2019). In addition, validation of non-invasive diagnostic tools such as MRE for its ability to quantify liver fibrosis in a larger patient population would also largely benefit the assessment of clinical trial outcome and longitudinal follow-up studies (Allen et al., 2020).

Furthermore, clinical relevance of a disease model can be further validated by proven efficacy of pharmacological interventions that have shown to be beneficial in clinical trials. Currently, pipeline drugs such as aramchol, GLP-1 receptor agonists, and cenicriviroc as well as OCA have all shown efficacy in improving NASH liver histology in NASH patients (Safadi et al., 2014; Armstrong et al., 2016; Friedman et al., 2018b; Eslam et al., 2019). The development of preclinical models can also leverage on clinical trial results where ideally, the use of these drugs in the animal model should display similar effects to those that are observed in the human studies. The converse is equally important, whereby drugs failing to show efficacy in human trials should ideally also fail in animal models that have both sensitivity and specificity.

Epidemiological studies indicate that the incidence of NAFLD and NASH is estimated to rise to 101 million and 27 million cases respectively by 2030 (Estes et al., 2018). Patients with progressed NASH have an increased risk of developing cirrhosis and liver failure, which is currently the second leading cause of liver transplantation (Wong et al., 2015). The enormous discrepancy between clinical trial results and preclinical data remain a prominent issue in the field of NASH research. The complexity of NASH disease pathology warrants the development of a clinically relevant NASH model for studying the mechanism of pathogenesis and drug evaluation.

## Author Contributions

CP and CXQ were responsible for the design and draft of the manuscript, RHR, OLW and AGS provided critical review and revision of the manuscript. All authors provide approval for publication of the content.

## Funding

This work was supported in part by the CASS Foundation (CXQ), Victoria Medical Acceleration Grant (RHR), and the Victorian Government's Operational Infrastructure Support Program. RHR was supported by the National Health and Medical Research Council (NHMRC) of Australia (ID1059960, ID1158013), and CXQ is Australia National Heart Foundation Future Fellow. Victorian Medical Research Acceleration Fund (RR, CXQ, AGS).

## Conflict of Interest

The authors declare that the research was conducted in the absence of any commercial or financial relationships that could be construed as a potential conflict of interest.
